# Dysregulation of coagulation in cerebral malaria

**DOI:** 10.1016/j.molbiopara.2009.03.006

**Published:** 2009-08

**Authors:** Christopher Alan Moxon, Robert Simon Heyderman, Samuel Crocodile Wassmer

**Affiliations:** a Malawi Liverpool Wellcome Trust Clinical Research Programme, College of Medicine, Chichiri, PO Box 30096, Blantyre 3, Malawi; b School of Clinical Sciences, UCD Building, Daulby Street, Liverpool L69 3GA, United Kingdom; c Liverpool School of Tropical Medicine, Pembroke Place, Liverpool L3 5QA, United Kingdom; d Department of Haematology, College of Medicine, University of Malawi, Private Bag 360, Chichiri, Blantyre, Malawi

**Keywords:** *Plasmodium falciparum*, Cerebral malaria, Coagulation, Haemostasis, Endothelium, Malaria

## Abstract

Cerebral malaria (CM) is a life-threatening complication of *Plasmodium falciparum* infection and represents a major cause of morbidity and mortality worldwide. The nature of the pathogenetic processes leading to the cerebral complications remains poorly understood. It has recently emerged that in addition to their conventional role in the regulation of haemostasis, coagulation factors have an inflammatory role that is pivotal in the pathogenesis of a number of acute and chronic conditions, including CM. This new insight offers important therapeutic potential. This review explores the clinical, histological and molecular evidence for the dysregulation of the coagulation system in CM, looking at possible underlying mechanisms. We discuss areas for future research to improve understanding of CM pathogenesis and for the development of new therapeutic approaches.

## 1 Introduction

Cerebral malaria (CM) is a severe form of *Plasmodium falciparum* infection that accounts for a large portion of the approximately 1 million acute malaria related deaths that occur annually. It is diagnosed clinically when a *P. falciparum* infected person is deeply unconscious with no other cause to explain the coma. It occurs in individuals who have not acquired *P. falciparum* specific immunity, with the burden being suffered overwhelmingly by children in sub-Saharan Africa. A conspicuous pathological feature is the adherence of *P. falciparum* infected erythrocytes (PfIE) in cerebral vessels [1]. However despite many years of research into its aetiology and treatment, mortality remains high and the pathogenesis is incompletely understood. In particular, the cause of death in this syndrome remains unclear.

Infection with *P. falciparum* is associated with systemic endothelial activation, patchy endothelial damage [2,3], low platelet counts, decreased levels of anticoagulants, generation of activated thrombin and pro-coagulant microparticles [4–8]. This strongly suggests a state of dysregulation of the coagulation system. Clinically haemorrhages are seen in the microvessels in the eye in most children with CM and these correlate with multiple microthrombi and haemorrhages in the microvessels of the brain in children who have died [9].

However gross clinical signs of coagulopathy are rare and traditional functional indices of coagulation – namely prothrombin time and activated partial thromboplastin time – are generally only mildly deranged. This leaves contention as to whether changes in coagulation factors play a role in pathogenesis or are merely a complication of other underlying pathogenic processes that manifest in a few individuals with severe disease.

Other reviews have described the interaction between coagulation and malaria [10–12], this article focuses on the clinical manifestations and biochemical features of dysregulation of coagulation in CM and explores the molecular mechanisms that might account for these findings. We propose a theoretical model for the role of coagulation in CM, discuss potential therapeutic options and provide suggestions for future research in this area.

## 2 Coagulation factors as inflammatory mediators and their role in sepsis

Normal haemostasis is a complex balance between pro- and anti-thrombotic pathways that take place on endothelial or platelet cell surfaces. This process involves the recruitment and tethering of several other cellular components including neutrophils and monocytes. Over the past 20 years, coagulation factors have emerged as important mediators of inflammation that demonstrate cross-talk with inflammatory cytokines [13]. These effects are involved in the pathogenesis of a number of acute and chronic inflammatory conditions. The signalling events initiated by thrombin, a central coagulation molecule, are the best characterised. Thrombin is capable of activating protease activating receptor 1 (PAR1), a G-protein-coupled receptor that, through modification of nuclear transcription factors, can stimulate a multitude of inflammatory events, including recruitment and activation of platelets and leukocytes and liberation of several pro-inflammatory cytokines [14].

It has become apparent that such inflammatory events may be initiated by coagulation molecules without overt clinical signs of a coagulopathy, such as bleeding or thrombi. A state in which coagulation is activated but balanced by regulatory pathways, termed compensated disseminated intravascular coagulation (DIC) [15], has been shown in several large prospective clinical studies to be predictive of poor outcome in bacterial sepsis in advance of clinical signs [16]. Further evidence of the importance of such a compensated state comes from therapeutic trials with anticoagulants. Treatment with infusions of the anticoagulants Antithrombin III (AT III) and activated protein C can attenuate mortality in animal models of bacterial sepsis [17,18] and activated protein C can improve outcome in placebo-controlled trials in humans, even in individuals with no overt clinical signs of coagulopathy [19]. Furthermore, activated protein C and AT III have anti-inflammatory effects and a protective effect on the endothelium *in vitro* [20]. While therapeutic trials of these factors in humans have not had the dramatic effect that was anticipated from animal trials, exciting new work on modified factors that influence inflammation without increasing risk of bleeding are in progress and may lead to the emergence of highly effective therapies for sepsis in the future.

There are both similarities and differences between the pathogenesis of *P. falciparum* infection and bacterial sepsis. The case for a potent malaria toxin, like lipopolysaccharide or lipoteichoic acid, remains controversial. Although the *P. falciparum* derived proteins glycosylphosphotidylinositol and hemozoin have been shown to have Toll-like receptor activity *in vitro* [21], it remains unclear how important these effects are *in vivo* [22]. *P. falciparum* malaria is an intravascular disease; whereas bacteria are able to traverse the endothelium into extravascular tissue, *P. falciparum* rarely exits the vessel lumen. Endothelial pathology occurs at sites of PfIE sequestration in the capillaries and post-capillary venules of end organs. Widespread endothelial dysfunction resulting in shock, like that seen in fulminant bacterial sepsis, is rare. However *P. falciparum* infection does result in the activation of the endothelium and in the release of pro-inflammatory cytokines [3,23]; mechanism implicated in the activation of coagulation in bacterial sepsis and so some similarities in the effects of both kinds of infection on the regulation of coagulation at the endothelial surface seem likely (Table 1).

## 3 Clinical manifestations of coagulopathy

### 3.1 Gross haemorrhage and thrombosis

While gross haemorrhagic or thrombotic events are uncommon in CM, their occurrence highlights a clear interaction between *P. falciparum* infection and coagulation. Overt bleeding is rare in children but in adults occur in approximately 5% of cases of CM, most commonly from the gastrointestinal tract [24]. Bleeding events have been described from other sites in association with CM, including rare severe bleeding episodes such as extensive bleeding into the skin associated with areas of necrosis called *purpura fulminans* [25,26], pulmonary haemorrhage [27] and intracranial bleeding [28–31]. Gross thrombotic events have also been described at post mortem in a few case reports; intracranially in the dural sinus [32] and in the lungs as thromboembolism [33]. Limb gangrene requiring limb amputation has also been described [34].

### 3.2 Microthrombi and fibrin deposits

Petechiae like lesions in the brain in fatal CM were first described nearly a century ago by Dudgeon and Clarke who recognised that the combination of thrombosis and haemorrhage seen in these patients might indicate that a coagulopathic state contributes to disease [35]. Recently detailed immunocytochemical studies using thrombin and platelet-specific antibodies have shown these lesions to be thrombi surrounded by ring haemorrhages as well as thrombi in intact vessels without haemorrhage (Wassmer, unpublished data). Among Malawian children with CM approximately 75% have thrombi [1]. These lesions are also seen in adults who have died from CM but are less common and tend to be fibrin deposits without platelets [2,36]. In fatal paediatric CM, brain thrombi and microhaemorrhages are confined to the capillaries in which PfIE sequester [1]. These children frequently also have thrombi in microvessels of other organs that show sequestration; namely the gut, lung, kidney, subcutaneous fat (Whitten RO, personal communication) and in the retina [9]. Thrombi are rarely seen in vessels without sequestration, implying a focal pathology that is mediated by interactions with PfIE.

Lesions seen at autopsy could be caused by peri-morbid events and might not be representative of pathology *in vivo*. However retinal haemorrhages have been shown to correlate strongly with haemorrhages in the brain at autopsy, but can also be seen in the majority of surviving patients with CM, implying that these lesions are a component of pathology in the acute phase of the disease [9,37,38]. Retinal haemorrhages have a central white area, where microthrombi have been shown by histology [9]. The number of haemorrhages seen at fundoscopy correlates with risk of death. These data show that in children with CM although gross coagulopathy is not common, microvascular coagulopathy occurs frequently and is associated with poor prognosis.

## 4 Mechanisms for the activation of coagulation

Malaria is an intravascular disease—the parasite does not invade the brain tissue or that of other target organs. The pathology of malaria in these tissues is therefore determined by the disruption of the normal intravascular environment. This may lead to the activation of the coagulation system in four different ways: (1) endothelial activation by pro-inflammatory cytokines; (2) circulating microparticles and activated platelets; (3) endothelial damage; (4) contact or binding between parasite-derived proteins on the PfIE surface and coagulation-based receptors on the endothelium or on circulating blood cells.

### 4.1 Endothelial activation

Endothelial activation in *P. falciparum* infection results in the upregulation of adhesion molecules on the endothelial surface that are implicated in PfIE adherence and pathogenesis: von Willebrand Factor (vWF), VCAM-1, ICAM-1, P-selectin and E-selectin [3,39–41]. This may occur indirectly via an intermediate factor that is affected by PfIE, for which inflammatory cytokines are the main candidate, or directly by interaction between parasite-derived molecules and endothelial receptors. A recent study performed in human volunteers demonstrated that vWF and vWF propeptide release from the Weibel–Palade bodies were significantly increased early in *P. falciparum* blood-stage infection, implying acute endothelial activation [41]. In African children with P. falciparum malaria, vWF levels were associated with severity, with the highest levels being observed in CM and non-CM severe malaria [39]. vWF is a large multimeric glycoprotein involved both in platelet adhesion and aggregation, and is produced predominately by activated endothelial cells. When released, vWF is in an activated conformation, which allows interaction with the platelet receptor gpIa/V/IV and triggers intravascular platelet aggregation [42]. This function primarily occurs under high shear stress conditions on the arteriolar side of the microcirculation [43]. One possible mechanism for increased vWF levels might be decreased activity of the vWF-cleaving protease ADAMTS13 (a disintegrin and metalloproteinase with a thrombospondin type 1 motif, member 13) and ADAMTS13 activity has been shown to be reduced in other conditions associated with thrombocytopenia and raised vWF levels, such as bacterial sepsis and thrombotic thrombocytopenic purpura [42]. However in early experimental malaria, raised vWF and thrombocytopenia were not accompanied by a change in ADAMTS13 activity [41]. These results suggest that a different mechanism for increased vWF levels may be implicated in malaria. The release of large multimers of vWF and adherent platelets into the circulation might provide multiple binding sites for PfIE and could potentially lead to major rheological disturbances by generating circulating clumps of vWF, platelets and PfIE. The possible association between an increase in vWF and mortality still remains to be investigated in adult and paediatric CM.

*In vitro*, endothelial nitric oxide (NO) decreases tissue factor (TF) expression [44] and is the major inhibitor of the exocytosis of Weibel–Palade bodies. It has recently been shown that endothelial NO is reduced in severe malaria in adults and is associated with increased concentrations of angiopoietin-2, an angiogenic factor also stored in Weibel–Palade bodies [45]. As vWF is copackaged with angiopoietin-2 in Weibel–Palade bodies, these results might explain the increase of vWF release in adult CM, leading to endothelial activation. While there is no published data in paediatric CM, it is entirely possible that a similar decrease in vascular NO availability occurs, leading to: (1) an increased expression of TF by microvascular endothelial cells, leading to an activation of the coagulation cascade and (2) a substantial augmentation in exocytosis of Weibel–Palade bodies with a subsequent increase of vWF release. Both effects could respectively explain not only the dysregulation of coagulation observed in paediatric CM, but also the characteristic acute endothelial activation inherent in this pathology.

The role of pro-inflammatory cytokines in the pathogenesis of *P. falciparum* malaria has been extensively studied. While several candidate cytokines for the activation of the coagulation system exist, TNF (formerly TNF-α) is the candidate with the best characterised role in CM [23,46]. PfIE can induce TNF production in several ways, the most important being either directly through the endothelium or via recruitment of monocytes [47]. In addition to its well known pro-inflammatory effects, TNF can also activate coagulation. It is a potent stimulator of TF, the major initiator of the coagulation cascade [48], which occurs through the activation of NFκB [49] and this effect is upregulated synergistically in the presence of thrombin via MAP kinase signalling [48]. TNF can also inhibit the activation of protein C by causing downregulation of thrombomodulin transcription [50].

### 4.2 Microparticles

Microparticles are vesiculations of the plasma membrane of eukaryotic cells, shed during physiological or pathogenic processes such as activation or apoptosis; they have been a topic of increasing interest in a number of disease states. Although many aspects of microparticle function are still unclear, a picture develops in which microparticles play an important role in inflammation, coagulation, and vascular homoeostasis. They have recently been shown to activate coagulation *in vitro* and to participate in thrombus formation in a model of vessel injury via their surface expression of TF and phosphatidylserine [51]. TNF is a potent agonist of endothelial vesiculation, leading to a dramatic increase in the production of endothelial microparticles. These microparticles display a pathogenic phenotype by expressing both cell adhesion and catalytic surface phospholipids able to trigger the extrinsic pathway independently of TF [52–54]. A recent study in Malawian children demonstrated significantly higher levels of circulating endothelial microparticles during the acute phase of CM compared to levels observed in parasitaemic controls. Levels of endothelial microparticles correlate positively with plasma levels of TNF and return to normal following recovery [5]. The enumeration of microparticles is carried out by flow cytometry and can be difficult due to their small size and the resolution of basic flow cytometers. However, fluorescence labelling allows microparticles to be differentiated from the background noise. In addition, a clean machine, filtered buffers and a proper dilution of the sample before analysis prevent a too high number of events passing through the laser and insure a better accuracy of the readings. It is not yet known if increased numbers of microparticles simply reflect an infection induced endothelial alteration or if they have a specific pathogenic role in CM.

Recently, analyses in a mouse model of CM [46] provided evidence for both pro-inflammatory and pro-coagulant effects of microparticles produced acutely in the plasma of CM susceptible mice. Indeed, microparticle-stimulated peritoneal macrophages were able to produce extremely high levels of TNF, reaching the production observed when stimulated by bacterial lipopolysaccharide, one of the most potent TNF inducers. In addition, microparticles were able to reduce significantly the clotting time of uninfected mouse plasma [55]. Although mouse models have been a significant resource in advancing knowledge of this disease, further investigations of the direct role of microparticles in human CM are needed.

### 4.3 Platelet adhesion to endothelium

Thrombocytopenia is an almost invariable feature of acute *P. falciparum* infection and appears to involve immune mechanisms, platelet activation, removal by the reticulo-endothelial system and consumption in different steps of coagulation [56]. The accumulation of platelets in cerebral microvessels in human CM was first described by electron microscopy [57], and more recently a *post mortem* study performed in Malawian children revealed that the accumulation of platelets in the brain was specific to children with a diagnosis of CM, as opposed to children who were parasitaemic but who had another cause of death [58].

Several studies performed with an *in vitro* model of the cerebral pathology showed that platelets can either adhere directly to the surface of TNF-stimulated endothelium via specific molecules such as platelet CD41 and endothelial CD40 [59], or adhere to vWF multimers at the surface of activated endothelium [39]. Having adhered to the endothelial surface, platelets are able to form bridges between PfIE and cerebral endothelium by providing new parasite receptors to brain endothelial cells initially devoid of them [59]. This phenomenon has been demonstrated *in vitro* and platelets lining the brain microvasculature between endothelial cells and PfIE have been described in a histopathological study of the brain of Malawian children who succumbed to CM [58]. Interestingly, PfIE can activate platelets *in vitro* by contact [60] leading to the transfer of TF stored in their alpha-granules to the platelet surface. Activated platelet surface TF may also derive from not only binding of TF-positive microparticles but also splicing of TF pre-mRNA and translation of the mature mRNA into protein. Indeed, human platelets express TF pre-mRNA and, in response to activation, splice this intronic-rich message into mature mRNA. Splicing of TF pre-mRNA is associated with increased TF protein expression [61]. Once at the cell surface, TF may require activation to reveal its pro-coagulant activity. Provided that this phenomenon occurs *in vivo*, it might lead to a dramatic platelet activation loop by initiating coagulation and promoting endothelial apoptosis, with both phenomena leading to an increase in platelet activation.

### 4.4 Endothelial damage and apoptosis

The endothelium acts as an envelope of uncharged cells devoid of TF, separating the blood from the negatively charged and TF-expressing connective tissue. Under normal physiological conditions this allows the blood to circulate without the activation of coagulation factors. When endothelial damage occurs, these circulating blood factors are exposed to both TF and a negative charged surface with resultant activation of coagulation through the TF pathway. While this mechanism is protective in cases of haemorrhage due to vascular trauma, it also contributes to the pathogenesis of conditions in which endothelial cells are altered through inflammation, apoptosis or hypoxia. It is implicated in the coagulopathies and organ dysfunction that occur in sepsis [62] and dengue haemorrhagic fever [63].

In mouse models of severe malaria several lines of evidence suggest that there is damage to the endothelium causing an increase in the permeability of vessels, particularly in the brain [46]. Surrogate markers for endothelial apoptosis are raised [64,65] and endothelial damage on retinal whole mounts of mice with experimentally induced CM has been described [64].

Such severe endothelial damage is rare in humans with *P. falciparum* infection but there is evidence to support a more subtle involvement of microvascular endothelial damage in the development of CM. Post mortem histological examination of cerebral microvessels by electron microscopy showed patchy areas of damaged endothelial cells amidst intact endothelium [2]. *In vivo*, assessment of retinal perfusion in patients with CM using angiography occasionally revealed multiple tiny areas of leakage, demonstrating focal areas of breakdown of the blood retinal barrier [66] which is structurally analogous to the blood–brain barrier [67]. However, extracellular oedema to imply gross blood retinal barrier breakdown is rare. Evidence that subtle endothelial damage occurs in a wider group of patients is also implied because levels of the endothelial bound receptor thrombomodulin is raised in peripheral blood, presumably because it is shed from the endothelial surface and levels are higher in patients with more severe disease [68].

One cause of endothelial damage that seems likely to be important in CM is apoptosis, which is also a specific cause of activation of coagulation [69]. The mechanism for apoptosis *in vivo* has not been established but *in vitro* PfIE adhesion on TNF-stimulated endothelial cells is strongly pro-apoptotic [70,71], an effect exacerbated by a prior co-culture with platelets [71]. This effect is mediated by the cytokine TGF-β released from alpha-granules of activated platelets [54]. Such a pathogenic mechanism may be important *in vivo* to promote endothelial apoptosis at sites of platelet–PfIE sequestration and in the presence of high levels of circulating TNF.

### 4.5 Receptor–ligand interactions between infected red blood cells and the host endothelium

Infection with *P. falciparum* leads to the expression of multiple parasite-derived proteins on the surface of the PfIE. Several families of these proteins have been described; they are highly variable, with coding regions generally located in sub-telomeric zones. Parasite-derived proteins interact with host receptors on the surface of the vascular endothelium and this interaction is a key factor in the pathogenesis. However, it has become apparent that these interactions may also have other important effects downstream. Binding of parasite-derived proteins on PfIE to dendritic receptors has been shown to prevent their maturation [72]. In addition, signalling events induced by PfIE adhesion can modify the surface expression of endothelial receptors [73,74]. The importance of low affinity interactions between parasite-derived proteins and endothelial receptors, including those that regulate coagulation, has received surprisingly little attention and yet the potential of these interactions seems large; both as PfIE roll along the endothelium prior to binding and during binding when the PfIE and its surface proteins are brought into direct apposition with the endothelium. Given the expression of several coagulation receptors at the endothelial surface, receptor–ligand interactions with PfIE may either interfere with the normal physiological interactions or induce signalling events that alter the regulation of coagulation. Although a specific ligand–receptor interaction has not been demonstrated, a signalling mechanism is implicated in the activation of TF when it is upregulated on the endothelial surface during infection, as described below. The thrombomodulin receptor involved in the activation of protein C and heparin sulphate, involved in the activation of AT III are other potential candidates since a portion of these molecules are binding sites for PfIE. The effects of these interactions on coagulation have not yet been explored.

## 5 Effect of *P. falciparum* on coagulation pathways

### 5.1 Activation of coagulation by tissue factor

TF is a transmembrane receptor expressed constitutively in most vessels in the cells under the endothelium. When it binds to factor VII, TF acts as the principal activator of coagulation. TF can be presented to factor VII as a result of endothelial damage, but also by circulating macrophages, platelets or microparticles or when it is upregulated at the endothelial surface. Many stimuli can induce such endothelial TF expression, including TNF [75], interferon gamma [76] and thrombin [77]. Upregulation of TF by lipopolysaccharide in bacterial sepsis leads to a reinforcing interplay between inflammation and coagulation [78]. In influenza and cytomegalovirus infections there is an upregulation of surface TF on monocytes, associated with both acute symptoms and an increased risk of atherosclerosis [79].

*In vitro*, PfIE can induce expression of surface TF on purified monocytes [80] and on cultured endothelial cells [49,81]. Although TNF is a candidate mediator, *in vitro* experiments suggest that PfIE induction of TF on endothelial cells is mediated by contact [81]. Interestingly, the upregulation of TF on endothelial cells is only induced by PfIE in their late life cycle, implying the involvement of an antigen that is only expressed in mature stages.

*In vitro*, PfIE can induce the expression of TF on purified monocytes [80] and on cultured endothelial cells [49,81]. While TNF is a candidate mediator, *in vitro* experiments suggest that PfIE induction of TF on endothelial cells is mediated by contact [81]. Upregulation of TF on endothelial cells is only induced by PfIE containing late-stage parasites, implying the involvement of an antigen that is only expressed by mature PfIE stages.

*In vivo*, the upregulation of TF has been associated with placental malaria and CM pathologies. In placental malaria, fibrin deposits impinging on the intervillous spaces are associated with the expression of TF on macrophages [82]. In CM, paediatric brain autopsy sections show TF expression on the endothelium of pre- and post-capillary vessels [81], a feature associated with thrombi and ring haemorrhages.

A drug developed to block TF activity which is a conjugate of the natural inhibitor of the TF pathway – tissue factor pathway inhibitor – reduced mortality in a primate model of sepsis [83], but did not improve outcome in a large intervention trial of sepsis in humans [84]. It has not been investigated whether this drug has any effect on outcome in severe malaria.

### 5.2 Protein C

The protein C pathway is a key modulator of both the inflammatory and haemostatic systems. Three main constituents of this pathway – protein C, protein S and thrombomodulin – have been shown to be affected in *P. falciparum* infection. *In vivo* evidence for the importance of the role of this pathway in the pathogenesis of infection has been demonstrated in Gram-negative sepsis, where a downregulation of protein C and thrombomodulin receptors on the endothelium are associated with microvascular thrombus formation in the dermis of children with meningococcal disease [85]. Furthermore activated protein C reduced mortality in a large double blind placebo-controlled trial of adults with severe bacterial sepsis, making it the only adjunctive treatment in sepsis of proven efficacy [19]. Consideration of activated protein C is of particular interest in malaria since in addition to its anticoagulant and anti-inflammatory effects it is also anti-apoptotic [20], protects endothelial barrier function [86] and reduces neuronal apoptosis secondary to ischemia [87]. Hence its actions might be protective against some of the underlying mechanisms associated with cerebral malaria pathogenesis.

Protein C and its cofactor, protein S, are vitamin K dependent plasma glycoproteins. Protein C is activated through the receptors thrombomodulin and endothelial protein C receptor (EPCR) when thrombin binds thrombomodulin. Activated protein C and S then form a complex that inhibits further thrombin production. Activated protein C also acts independently as an anti-inflammatory agent [88]. Hence the activation of protein C acts like a molecular switch in which the pro-coagulant, pro-inflammatory factor thrombin triggers the availability of an anticoagulant, anti-inflammatory factor.

*In vivo* plasma protein C antigen and activity [4,6,8] are decreased in *P. falciparum* malaria infection. Levels show an inverse correlation with clinical severity [6] and parasite density [4] and improve with treatment, correcting to within normal range in convalescence. Similarly protein S levels tend to be lower acutely in patients with severe but not mild *P. falciparum* infection and are lowest in patients with severe complications [6,7]. These studies were predominately in adults and including few patients with CM. We would expect the effects on these factors to be more pronounced in CM but studies are needed to investigate this particularly in African children.

The effectiveness of activated protein C as a treatment has not been systematically investigated. Case reports of patients with severe *P. falciparum* malaria infection improving following treatment with activated protein C [89,90] are insufficient to prove safety or efficacy in malaria at present.

### 5.3 Thrombomodulin

Activation of protein C is initiated by binding of thrombomodulin and decreased activation of protein C in bacterial sepsis has been shown to relate to downregulation of thrombomodulin. These factors point to thrombomodulin as a primary candidate to explain the decreased activation of protein C in *P. falciparum* infection. Thrombomodulin is an integral membrane glycoprotein expressed on nearly all endothelial tissue although notably only at low levels in the brain [91]. A side chain of thrombomodulin, chondroitin sulphate A (CSA), increases the anti-thrombotic properties of thrombomodulin 10–20-fold [92] and this side chain can bind PfIE [93]. While the importance of PfIE binding to thrombomodulin in sequestration is unclear, low affinity interaction with the thrombin binding site and therefore the activation of protein C could lead to a decreased capacity to buffer thrombin, although this mechanism is yet to be explored. Decreased availability of thrombomodulin may also occur due to raised TNF, which can induce a downregulation of thrombomodulin on cultured endothelium [94], or by shedding of thrombomodulin from the endothelium, which can be induced *in vitro* using serum from *P. falciparum* infected patients [68].

### 5.4 Antithrombin III

AT III is another major endogenous anticoagulant and anti-inflammatory agent [95]. It is a circulating glycoprotein that can inhibit thrombin directly and via a number of upstream factors. AT III has poor anticoagulant activity until activated, principally by heparin sulphate.

Like protein C, AT III plays an important role in the aetiology of sepsis with levels being negatively associated with severity and outcome [96]. Infusion of AT III prevents mortality in an animal model of sepsis [17] and has shown some success in placebo-controlled trials in humans with DIC and sepsis [96].

Levels of AT III antigen [7,8] and activity [6] are decreased in severe *P. falciparum* infection. Levels correlate with severity and there is rapid recovery following antiparasitic treatment [6]. Low antigen levels are likely to be due to increased consumption from thrombin binding in thrombin–antithrombin (TAT) complexes as TAT complexes are significantly increase in severe malaria [4,8]; suggesting AT III is ‘mopping up’ excess thrombin. Decreased activity may reflect a decreased availability of antigen or may reflect compromised activation by heparin sulphate. Since heparin sulphate is a major binding site for parasite-derived proteins [97], a possible mechanism for the latter is that PfIE ligands interact with heparin and compromise the heparin dependent activation of AT III.

Since heparin can also disrupt rosette formation [98] and inhibits parasite invasion and growth [99,100], heparin like drugs are potential therapies and in a simian model of malaria, treatment with heparin resulted in a decrease in mortality [101]. The only controlled studies to look at heparin treatment in humans [102,103] were not powered to detect an improvement in outcome and World Health Organization guidelines do not recommend the use of heparin in malaria because of the potential to induce haemorrhage [104]. Heparinoids such as curdlan sulphate that disrupt PfIE binding but that have a lower risk of causing bleeding may represent an alternative. In two small studies, although not powered to show effect on mortality, curdlan sulphate decreased time until clearance of fever with no noted side effects and the beneficial effect was most noted in individuals with CM [105].

## 6 Theoretical model and possible reasons that haemorrhage occurs more in the brain

**Fig. 1:**
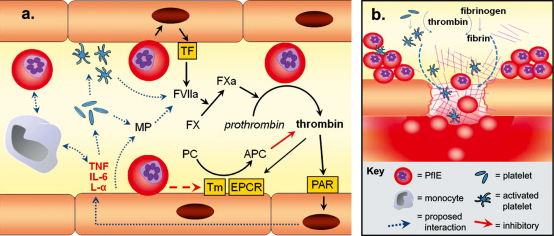
Theoretical model for the dysregulation of the coagulation system in cerebral malaria. (a) Early in infection the coagulation system is activated by a combination of the host immune response and parasite-derived protein interactions with host endothelium, and circulating cells. This leads to: (1) the generation of thrombin and intracellular signalling through PAR; (2) the activation and recruitment of platelets and monocytes; (3) the vesiculation of microparticles from the endothelium and platelets; (4) an excessive visibility of TF on the following activated components: endothelium, platelets, monocytes and microparticles. In most patients this system is balanced by the protein C and AT III pathways (latter not pictured here). However, at the most severe end of the spectrum these pathways are impaired by intense sequestration of PfIE within the microvasculature (b). This sets up a potential positive feedback cycle of inflammatory and coagulation events. Thrombin promotes the conversion of fibrinogen to fibrin which adheres to activated platelets to form thrombi and is itself pro-inflammatory. Excessive inflammation at these sites from a combination of inflammatory events and cytokines, unbuffered by anticoagulants, and activation of apoptosis by PfIE contact and TGF-β, leads to endothelial damage and loss of tight junction function. This creates gaps in vessels and, in the context of excessive local consumption of coagulants by the thrombi, leads to microhaemorrhages. L-α: lymphotoxin-α; IL-6: interleukin-6.

Using available evidence we propose a model for the involvement of dysregulation of coagulation in the pathophysiology of CM in which intense sequestration in microvessels leads to a reinforcing cycle of inflammatory and coagulant events resulting in focal endothelial damage, thrombi and microhaemorrhage (Fig. 1). Inflammatory events are also likely to increase sequestration through upregulation of receptors on the endothelial surface such as ICAM-1.

The observation that thrombi and haemorrhage occur to a greater extent in the brain than in other organs in which parasites sequester requires further consideration. It cannot be explained purely by the degree of sequestration, as sequestration is not significantly higher in the brain than in other organs in which thrombi and haemorrhage are less common [106]. The implication is therefore that cerebral microvessels are somehow predisposed to decompensation. One possible explanation is the low levels of thrombomodulin expression in vessels in the normal human brain [91] and the low levels of EPCR found in microvessels. The potential for cerebral microvessels to activate protein C would therefore be low, even in the absence of pathology. These vessels would then be prone to decompensation resulting in thrombi and haemorrhage earlier than in other organs. Another hypothesis is that the distinct expression pattern of endothelial receptors in the brain, which leads to a different profile of PfIE-receptor binding – such as an increased tendency to bind ICAM-1 in the absence of CD36 receptors – also leads to qualitatively different PfIE–endothelial interactions. This may be associated with different resulting signalling events more likely to result in conditions that favour haemorrhage. A better understanding of the specific interactions between PfIE and the coagulation and inflammatory system, particularly at the endothelial level will help guide our understanding of why cerebral endothelium might be affected differently to endothelium in other organs.

## 7 Adaptive responses to minimise coagulopathy

Given the degree of interaction between PfIE and the endothelium and the extremely high levels of parasitaemia observed in many children with CM, some with >40% of erythrocytes being infected, it is surprising that coagulation is not more dysregulated. However this can be considered in the light of a long evolutionary history of human infection by this parasite. The evolutionary pressure of *P. falciparum* on the human host is probably the most significant of any parasitic interaction [107] and this has resulted in a number of genetic mutations to confer protection, such as sickle cell anaemia and thalassemia. Similar pressure was applied by the host on the parasite and has led to the development of elaborate mechanisms for evading the host immune system. Induction of thrombi or haemorrhage is deleterious to the host because it alters blood supply to vital organs and deleterious to the parasite as parasites in PfIE consumed in thrombi or that leak out of the vessel by haemorrhage are unable to then infect new erythrocytes. A hypercoagulable state would have dramatic effects for both organisms; it is therefore most likely that both organisms would have developed mechanisms to minimise the activation of the coagulation system.

Several host factors produced in response to infection are protective against inflammatory damage. Among them, erythropoietin [108] may be raised up to 30-fold in CM and reaches levels associated with protection against inflammation and tissue damage in stroke. Parasites can also exert a protective effect on the endothelium. Indeed, it has been reported that in some cases PfIE but not uninfected erythrocytes induce *in vitro* a downregulation of TNF-associated apoptosis in a contact dependent manner [109]. It seems highly plausible that there are many other host and parasite mechanisms that protect against activation of the coagulation system both downstream by limiting inflammation and directly. Further investigation of these mechanisms is required not only to improve our understanding of parasite–host interactions but also to provide new therapeutic approaches for CM.

## 8 Suggestions for future research

The majority of published work on the coagulation system in malaria comes from studies on soluble factors and on traditional coagulation assays. In trying to understand the role of coagulation in CM, these studies have several limitations: (1) they are predominately carried out in adult populations and are from areas of low or inconstant transmission of *P. falciparum* and therefore are not representative of the paediatric group who suffer the major burden of disease in sub-Saharan Africa; (2) the number of patients with severe disease in these populations is small and there are no studies specifically on CM; (3) they are mainly in study populations that are epidemiologically poorly characterised and (4) severe malaria is investigated as a heterogeneous group without distinguishing malarial syndromes, which may have different aetiologies. In addition, recent studies have led to the development of new assays [110] and to a consensus scoring system that allows more consistent characterisation of a pro-coagulant state [15] and it has been shown that these tools enable prediction of outcome in advance of clinical signs [16]. Investigation of plasma factors in a well characterised group of African children with CM that incorporate these developments is warranted.

However factors in blood can only give a downstream picture of the regulation of coagulation, which occurs principally at the endothelial surface. Studies of endothelial events have demonstrated changes in the endothelial expression of coagulation receptors in sepsis and in neoplastic disease that have shed light on their underlying pathology [85,111]. But there has been little published on the changes to endothelial coagulation receptors in malaria. Looking at such local events is particularly relevant in a disease like *P. falciparum* malaria in which disease occurs principally in microvessels; since focal events here might be important locally in end organs but not be detectable in peripheral blood. To accurately understand the contribution of coagulation to CM pathology we must therefore be able to explore endothelial events in the small vessels of affected tissues. One means of doing this is to look at microvessels in post mortem tissue. This has the advantage of allowing the exploration of endothelial events in several different organs in one individual and we have discussed interesting work on TF expression that has used this method [81]. However information from patients post mortem only provides information on advanced disease and includes events that may only occur in the agonal or post mortem period. A group has looked at studying endothelial receptor expression in skin biopsies *ex vivo* but found that events in skin microvessels were poorly representative of the extent of disease [112], perhaps because there is minimal sequestration in this tissue. Development of a representative *ex vivo* model would be invaluable.

Endothelial events can also be explored *in vitro* by using cultured endothelial monolayers and parasites to explore host–parasite interactions. Models exist for studying such interactions under flow conditions to mimic the sheer forces encountered in the intravascular environment. Studies using such techniques have given us valuable insights into disease mechanisms both in terms of adhesive and signalling events, and could be used to look at coagulation-based events in CM.

## 9 Conclusion

Although obvious thrombosis or haemorrhage have been considered rare in *P. falciparum* infections, detailed post mortem studies and examination of the retina *in vivo* have revealed that microvascular lesions are common in children with CM. A number of coagulation related molecular and cellular events underlie these changes and are apparent even in patients with less severe disease. Given the importance of coagulation factors as regulators of inflammation these events are likely to play an important role in pathogenesis. However there is a paucity of research directed specifically at CM or at endothelial phenotype in malaria and there remain significant gaps in our understanding of the exact nature and mechanisms responsible for these changes. Advances in this area may play an important role both in our understanding of CM pathogenesis and also in the development of new therapies and prognostic tools.

## Figures and Tables

**Table 1:** Description of some key coagulation molecules.

Molecule	Function	SF or TMR	Site of expression	Effect in *P. falciparum* malaria
TF	Main activator of coagulation cascade	TMR	Expressed in all vessels on underlying cells of basal lamina and the basement membrane. Expressed on *activated* endothelial cells, monocytes, platelets and microparticles	*Severe malaria:* NI
				*CM:* TF expression on endothelium in cerebral vessels in CM and associated with thrombi [81]
				*Mechanism:* PfIE cause upregulation of TF on endothelium *in vitro* in contact dependant manner [49,81]

Thrombin	Converts fibrinogen to fibrin. Inflammatory mediator via PAR1 signalling	SF	Produced from prothrombin on activation of coagulation. Very rapidly inactivated and therefore difficult to measure directly	*Severe malaria:* Raised TAT complexes [4,6,8] and Fibrinopeptide A [113] indicate increased thrombin generation. Correlate with severity of infection [4,6]
				*CM:* NI

Protein C	Inhibits production of thrombin and anti-inflammatory when activated	SF	Circulates in inactive form until activated by thrombomodulin and EPCR initiated by the binding of thrombin to thrombomodulin	*Severe malaria:* Decreased antigen and activity [4,6,7]
				*CM:* NI

Thrombomodulin	Inhibits thrombin by binding and involved in activation of protein C	TMR	Expressed on all vascular endothelium although at a lower lever in the brain. Cleaved from the membrane to become a soluble factor when there is endothelial damage	*Severe malaria:* Circulating thrombomodulin increased [68]; effect on endothelial thrombomodulin expression not reported
				*CM:* NI

EPCR	Involved in activating protein C by stabilising the interaction of protein C with the thrombin–thrombomodulin complex	TMR	Expressed on all vascular endothelium; lower level in microvessels	NI

AT III	Inhibits thrombin both directly and upstream	SF	Activated by heparin sulphate	*Severe malaria:* Antigen and activity levels decreased [6,7]
				*CM:* NI

Fibrin	Fibrin strands form a mesh that associates with platelets to form a clot. Also pro-inflammatory	N/A	Produced from fibrinogen in the presence of thrombin. Once produced forms clumps and can only be measured by surrogate markers of its breakdown	*Severe malaria:* Evidence for increased fibrin generation in some studies [101,114] but ambiguous
				*CM:* Deposits of fibrin or thrombi in vessels in diseased organs [1,2,9]

SF: soluble factor; TMR: trans-membrane receptor; NI: not investigated.
